# Acute Hemodynamic Impact of Transcatheter Edge-to-Edge Repair in Hypertrophic Cardiomyopathy

**DOI:** 10.1016/j.jscai.2023.100635

**Published:** 2023-04-04

**Authors:** Ahmad A. Al Turk, Akram W. Ibrahim, Marvin H. Eng

**Affiliations:** a Division of Cardiology, Department of Medicine, The University of Arizona School of Medicine, Phoenix, Arizona; b Division of Cardiology, Department of Medicine, Arizona Heart Institute, Abrazo Health System, Phoenix, Arizona

**Keywords:** transcatheter edge-to-edge repair, MitraClip, hypertrophic cardiomyopathy

Treatment options for hypertrophic cardiomyopathy (HC) have evolved since Morrow’s first myotomy in 1961.^1^^,^^2^ The MitraClip device (Abbott) has been used as an adjunct to alcohol septal ablation (ASA) or as a single intervention in patients ineligible for septal reduction therapies.^3^ Herein, we describe a unique case of transcatheter edge-to-edge repair (TEER) in a patient who agreed to undergo TEER over ASA. Hemodynamic waveforms were recorded at rest and with dopamine infusion at 5 μg/min. Pressure was simultaneously recorded using a 4F pigtail catheter and an 8F sheath positioned in the left ventricle and aortic arch, respectively. Provoked left ventricle outflow tract (LVOT) gradient was recorded of the beat following an induced premature ventricular contraction. A premature ventricular contraction results in an augmented inotropic response of the following cardiac cycle, hence accentuating the gradient in patients with dynamic LVOT obstruction.^4^

## Case report

An 82-year-old man with symptomatic HC despite medical therapy, systolic anterior motion of the mitral valve, and severe posteriorly directed mitral regurgitation (MR) jet was referred for discussion of percutaneous treatment options. The patient was considered at high risk for surgical intervention, and he agreed to undergo TEER over ASA.

**Figure 1:**
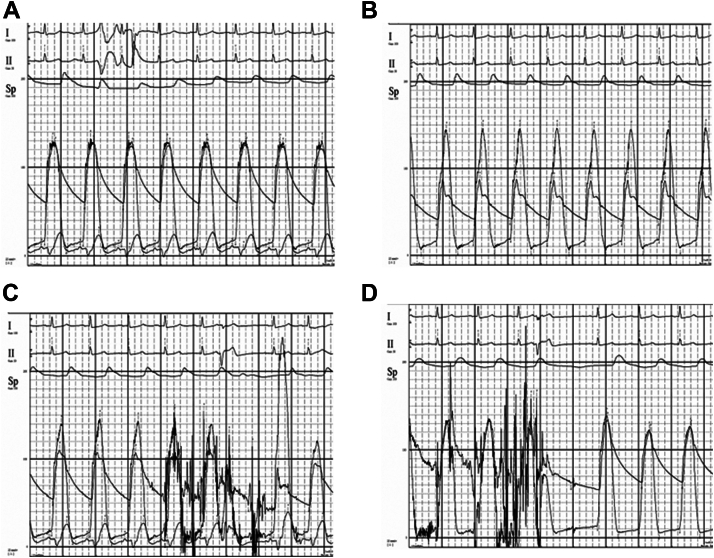
**Hemodynamic waveforms.** (**A**) No significant LVOT gradient at baseline. (**B**) LVOT gradient ∼60 mm Hg with dopamine infusion. (**C**) Provoked LVOT gradient >120 mm Hg. (**D**) Provoked LVOT gradient <5 mm Hg after MitraClip implantation. LVOT, left ventricle outflow tract.

The patient underwent TEER with a MitraClip positioned at the medial aspect of A2-P2. This resulted in reduction of MR to a trace level and elimination of provoked LVOT gradient (Figure 1A-D). The patient reported symptom resolution on subsequent evaluation.

## Discussion

Identifying and targeting the etiology of patients’ symptoms should be the focus when evaluating a patient with HC. Although ASA is an appropriate first step in patients with LVOT obstruction related symptoms, TEER has an emerging role when symptomatic MR is the predominant manifestation. When symptoms are mixed or when symptom etiology is unclear, patients may benefit from both ASA and TEER but perhaps in a stepwise fashion. Hemodynamic evaluation is essential in patients with HC for guiding therapy and assessing treatment response.

## Conclusion

When assessing patients with HC, effort should be made to identify the etiology of symptoms as this may favor one treatment option or the other. In our case, treatment of MR resulted in symptom resolution despite persistent basal septal hypertrophy.

**Pearls in Hemodynamics from editors Larry S. Dean, MD, and Morton J. Kern, MD**

• Hypertrophic cardiomyopathy frequently presents with complex physiology, which includes not only the septum but the mitral valve.
• “Removing” the anterior mitral valve motion with TEER may result in improved hemodynamics of both the outflow tract and mitral valve.
• In patients with a mixed hemodynamic picture, a stepwise approach may result in improvement in symptoms without the need for septal ablation.
